# Too Demanding to Stay? Fatigue and Depressive Symptoms as Mediators of Turnover Intention in Romanian Anaesthesia and Intensive Care Nurses: A Cross-Sectional Study

**DOI:** 10.3390/nursrep16080264

**Published:** 2026-07-30

**Authors:** Doina Carmen Mazilu, Viorica Nedelcu, Ilona Voicu, Ioana Grigoraș

**Affiliations:** 1 The Order of Nurses, Midwives and Medical Assistants in Romania—Bucharest Branch, JBI Romanian Centre for Nursing Research, 12 Avrig Street, District 2, 021575 Bucharest, Romania; mazilu.carmen@oammrbuc.ro (D.C.M.); ilona.voicu@oammrbuc.ro (I.V.); 2 The Faculty of Midwifery and Nursing, “Carol Davila” University of Medicine and Pharmacy, 8 Eroii Sanitari Avenue, District 5, 050474 Bucharest, Romania; 3 The School of Medicine, “Grigore T. Popa” University of Medicine and Pharmacy, 16 Universității Street, 700115 Iași, Romania; ioanagrigoras.ro@gmail.com

**Keywords:** nurses, job demands, turnover intention, professional fatigue, depressive symptoms

## Abstract

**Background/Objectives:** Nurses working in Anaesthesia and Intensive Care Units (AICUs) face high job demands that may negatively affect their well-being and increase turnover intention. In healthcare systems experiencing workforce shortages, such as Romania, understanding these mechanisms is essential for developing effective retention strategies. This study examined the relationship between job demands and turnover intention among Romanian AICU nurses, focusing on the mediating roles of professional fatigue and depressive symptoms. **Methods:** A cross-sectional survey was conducted among 1001 nurses from the AICUs of 68 Romanian public hospitals. Job demands were measured using a composite index including night shifts, long working hours, schedule changes, exposure to aggressive patients, calls from home, and alarm-related stress. Professional fatigue was assessed as subjective exhaustion after work, depressive symptoms with the Patient Health Questionnaire-9, and turnover intention as the intention to leave the workplace within 12 months. Mediation analyses were performed using PROCESS macro (Model 4) with logistic regression. **Results:** Job demands were positively associated with turnover intention. Higher job demands predicted increased professional fatigue and depressive symptoms, both significantly associated with turnover intention. Mediation analyses demonstrated significant indirect associations through both professional fatigue and depressive symptoms, indicating partial mediation. **Conclusions:** Job demands are related to turnover intention among Romanian AICU nurses both directly and indirectly through professional fatigue and depressive symptoms. Retention strategies should address not only workload reduction, but also prevention and coping interventions targeting fatigue and psychological distress.

## 1. Introduction

### 1.1. The Romanian Healthcare Context

The Romanian public healthcare system has been facing a series of challenges, including ensuring the necessary human resources to meet its needs, especially in public hospitals. The National Health Strategy 2023–2030 highlights that the distribution of healthcare professionals across specialties and competencies does not adequately meet the health needs of the Romanian population [1]. At the same time, European Commission reports describe a paradoxical situation: Romania trains a large number of healthcare professionals, yet continues to experience substantial workforce shortages because of migration [2].

Public hospitals are the most affected facilities. In the last two decades, the migration of doctors and nurses to the healthcare systems of other European countries was increasingly dramatic. For the Western European healthcare markets, Romania functions as one of the “source countries and thus faces a critical shortage of qualified medical personnel.” [3]. In return, the deprivation of doctors, nurses and other qualified personnel leaves Romanian public hospitals severely understaffed and structurally weakened.

If ten years ago the main causes of migration were represented by economic factors, especially income level, at present, organizational and psychological aspects play an increasingly important role in the decision of the medical personnel to leave: “A significant factor in the decision to leave the Romanian healthcare system is at a psychological level, which is mainly observed through the high level of stress reported by the healthcare personnel. Thus, the very high workload under extreme pressure, combined with the shortage of personnel, can lead, in certain situations, to burnout and professional exhaustion” [3].

Therefore, nursing staff retention becomes an issue that requires not only measures at the macro level, but also better management at the micro level in medical facilities. Thus, organizational elements can contribute to the mitigation of professional stress and fatigue. Identifying the factors related to turnover intention are highly relevant and useful in drafting more effective policies for motivating and retaining nurses, especially in workplaces which require a high level of resilience.

### 1.2. Turnover Intention, Job Demands and Psychological Well-Being

Turnover intention, defined as an employee’s conscious and deliberate intention to leave the current organization, is widely recognized as one of the strongest predictors of actual turnover [4,5]. In nursing, turnover intention has important consequences not only for workforce stability but also for quality of care, patient safety, organizational costs, and continuity of healthcare services. Consequently, identifying the mechanisms that contribute to turnover intention has become a major research priority.

Professional fatigue has been described as a multidimensional condition resulting from prolonged exposure to excessive work demands, work environments, and work schedules that impair employees’ physical, cognitive, and emotional functioning. (Throughout this manuscript, the term *professional fatigue* is used consistently to refer to nurses’ perceived work-related fatigue, operationalized as the subjective feeling of exhaustion experienced after a working day. The terms “occupational fatigue”, “work-related fatigue”, and “subjective exhaustion” are therefore used conceptually to describe the same occupational phenomenon, although the study variable is referred to as *professional fatigue* throughout the manuscript.) [6].

Previous research consistently demonstrates that nurses working in highly demanding clinical environments experience increased occupational stress, professional fatigue, and mental health problems, which contribute to a greater likelihood of leaving their jobs [7]. Anaesthesia and Intensive Care Units (AICUs) represent some of the most demanding clinical settings. Nurses are continuously exposed to critically ill patients, advanced medical technology, shift work, emergency situations, frequent alarms, and emotionally challenging interactions with patients and their families. These working conditions require sustained cognitive, physical, and emotional effort, increasing vulnerability to professional fatigue and psychological distress [8]. Similarly, recent evidence from the World Health Organization (WHO) survey of healthcare professionals across the European Union, Iceland and Norway indicates that nurses experience substantially higher rates of depressive symptoms than the general population. Often, this poor mental health is connected with stronger intentions to leave the profession [9].

Taken together, these findings suggest that excessive job demands are associated with workforce retention not only directly but also indirectly through their negative effects on physical and psychological well-being. Nevertheless, the mechanisms linking occupational demands, professional fatigue, depressive symptoms, and turnover intention remain insufficiently understood, particularly in highly specialized nursing settings.

### 1.3. Theoretical Framework

The present study was guided by the Job Demands–Resources (JD-R) Model [10] and Affective Events Theory (AET) [11]. According to the JD-R Model, excessive job demands activate a health-impairment process that may result in professional fatigue, psychological strain, and an increased intention to leave the organization. Consistent with this framework, recent evidence suggests that the relationship between job demands and turnover intention is explained not only by direct effects but also by psychological mediating mechanisms [12].

AET proposes that workplace experiences trigger emotional responses that subsequently are connected with employees’ attitudes and behaviors. From this perspective, persistent exposure to demanding working conditions may contribute to depressive symptoms, which, in turn, increase turnover intention.

The two theoretical frameworks are complementary rather than alternative. The JD-R Model explains how structural characteristics of the work environment, particularly high job demands, initiate a health-impairment process leading to reduced physical and psychological resources. AET extends this perspective by explaining how these demanding work experiences are translated into emotional and psychological responses, such as professional fatigue and depressive symptoms, which are associated with employees’ attitudes and behavioral intentions, including turnover intention. Together, these models provide a comprehensive framework linking organizational stressors, psychological responses, and nurses’ intention to leave their workplace.

### 1.4. Research Gaps and Study Contribution

Despite the growing body of research linking job demands to nurses’ turnover intention, important knowledge gaps remain.

First, although professional fatigue and depressive symptoms have each been associated with turnover intention, relatively few studies have examined these variables simultaneously as complementary mediating mechanisms through which job demands may influence nurses’ intention to leave.

Second, most available evidence originates from Western Europe, North America, and Asia, whereas evidence from Eastern European healthcare systems remains scarce. Finally, because AICUs represent one of the most demanding nursing specialties, findings from general nursing populations cannot be assumed to apply directly to this clinical setting.

Building upon previous evidence and addressing these identified research gaps, the primary aim of this study was to examine the association between job demands and turnover intention among Romanian AICU nurses and to investigate whether this association is statistically mediated by professional fatigue and depressive symptoms. Specifically, the study examined both the direct association between job demands and turnover intention and the indirect associations through the two proposed mediators.

The present study was designed to address these gaps by examining the association between job demands and turnover intention among Romanian AICU nurses while simultaneously testing the mediating roles of professional fatigue and depressive symptoms. In doing so, the study contributes to the existing literature in three ways. First, it simultaneously examines two complementary psychological mediators within a single analytical model. Second, it focuses on AICU nurses, a specialty characterized by exceptionally high occupational demands but relatively limited empirical evidence. Third, it extends the international literature by providing evidence from Romania, an Eastern European healthcare system facing persistent workforce shortages and high migration of healthcare professionals.

Taken together, the JD-R Model and AET provide the conceptual rationale for hypothesizing that professional fatigue and depressive symptoms statistically mediate the association between job demands and turnover intention:

**Hypothesis** **1 (H1).** *Job demands are a predictor of turnover intention among AICU nurses.*

**Hypothesis** **2 (H2).** *Professional fatigue (M1) and depressive symptoms (M2) are hypothesized to statistically mediate the association between job demands and turnover intention. Specifically, higher job demands are expected to be associated with greater professional fatigue and more severe depressive symptoms, which, in turn, are associated with higher turnover intention.*

**Figure 1:**
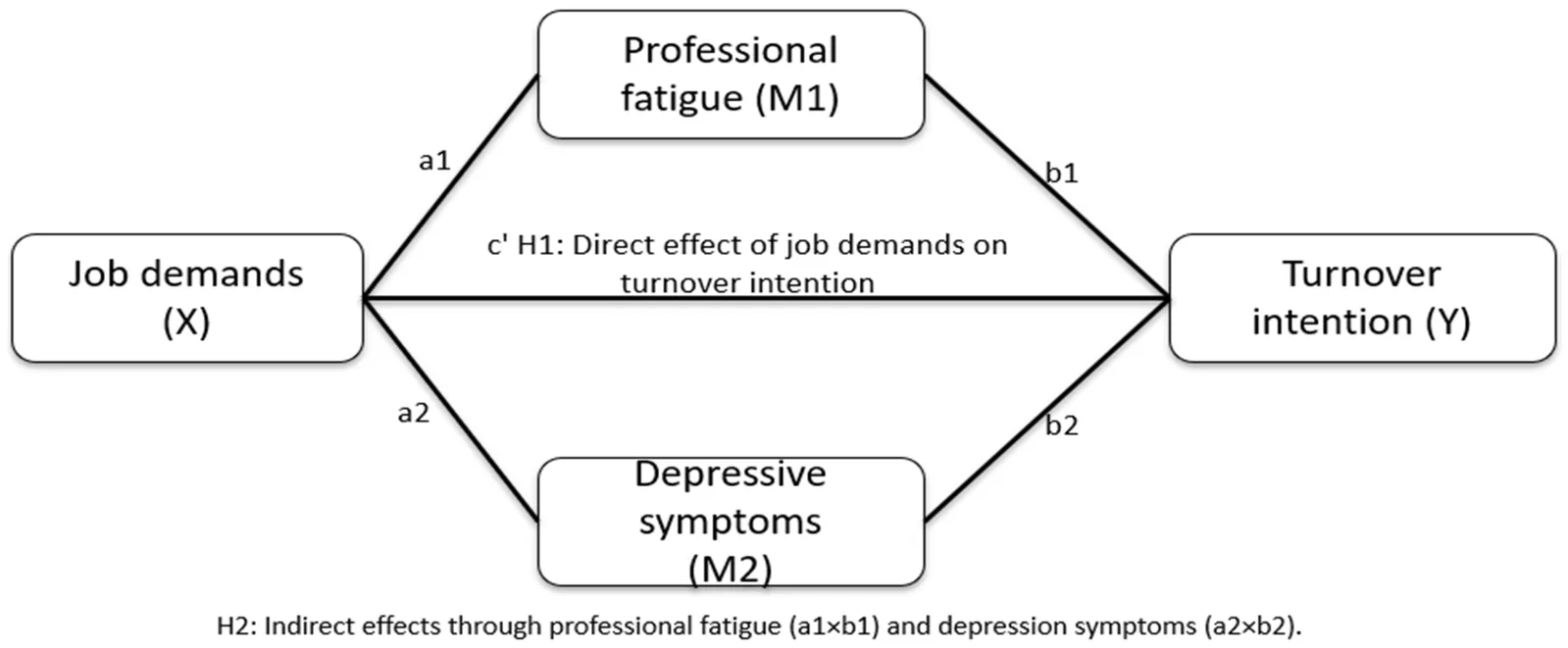
Conceptual model of the hypothesized relationships among job demands, professional fatigue, depressive symptoms and turnover intention.

The conceptual model tested in the present study is presented in Figure 1.

## 2. Materials and Methods

### 2.1. Study Design

The study was conducted within the framework of the partnership established in September 2024 between the Order of Nurses, Midwives and Medical Assistants in Romania (OAMGMAMR) and the European Patient Safety Foundation (EUPSF), together with the launch of the *Fighting Fatigue Together* campaign in Romania [13].

Thus, the present research represents a sub-study of a broader national project investigating professional fatigue among Romanian nurses and midwives.

### 2.2. Questionnaire Development

The primary objective of the project was descriptive and exploratory, aiming to provide a comprehensive assessment of professional fatigue and its organizational, occupational, and psychological correlates across Romanian healthcare settings. Accordingly, the questionnaire was designed to capture a wide range of dimensions relevant to nurses’ working conditions rather than to provide an in-depth assessment of a limited number of constructs.

Prior to the national survey, the questionnaire was pilot-tested in a sample of 283 nurses and midwives from six public hospitals in Bucharest. The pilot study was conducted online, using a Google Form questionnaire. In addition to assessing clarity and comprehensibility, participants’ feedback and response distributions were reviewed to identify problematic items and response options, leading to minor revisions of wording, response categories, and questionnaire formatting before the final online version was implemented. Ambiguous or problematic wording was refined, response options were revised where necessary, and response categories were developed for selected open-ended questions according to the frequency distribution of participants’ answers. In addition, for the multi-item scales included in the questionnaire, internal consistency was evaluated using Cronbach’s alpha coefficients.

Because the survey was administered online to a large national sample, minimizing respondent burden and maximizing completion rates were important methodological considerations. Therefore, when appropriate for the descriptive objectives of the project, brief global indicators were preferred over lengthy multi-item instruments. Although multi-item scales generally provide a more comprehensive assessment of complex constructs, single-item measures may be acceptable for global ones in special circumstances [14], such as when there is a necessity to minimize respondent burden, particularly in large-scale surveys administered online.

Consequently, professional fatigue and turnover intention were assessed using single-item measures considered appropriate for capturing global perceptions and behavioral intentions within the context of this nationwide descriptive survey. In contrast, depressive symptoms were assessed using the validated Patient Health Questionnaire (PHQ-9), given its central role in the mediation model examined in the present study. For the purposes of this study, professional fatigue was operationalized as an overall subjective perception of current work-related fatigue rather than as a multidimensional construct. Accordingly, a global single-item measure was considered appropriate for capturing participants’ perceived level of professional fatigue.

The original Romanian Version of the questionnaire is available as Supplementary Material S1.

### 2.3. Participants and Data Collection

The survey invitation was disseminated by the OAMGMAMR through its county branches and the Bucharest branch. These branches subsequently forwarded the invitation and survey link to healthcare institutions within their jurisdiction, where Directors of Nursing were asked to distribute the online questionnaire to eligible nurses and midwives. Directors of Nursing acted solely as intermediaries for distributing the survey invitation and had no access to participants’ responses or information indicating who chose to participate. Survey responses were submitted directly through the anonymous online questionnaire. Participation was entirely voluntary, based on a convenience sampling approach, and choosing not to participate had no consequences for employment or professional standing. Hospital identifiers were intentionally omitted from the questionnaire to minimize the risk of participant identification, particularly within small clinical units. Participant eligibility was determined by self-report, and the study invitation specified the eligibility criteria. To minimize duplicate or multiple submissions, the Google Forms questionnaire was configured using the “Limit to 1 response” setting, allowing only one submission per participant. Most questionnaire items used to construct validated scales or composite variables were configured as required fields in the online questionnaire. However, the individual items later used to derive the cumulative Job Demands Index were not configured as required fields in the original nationwide survey.

Because the survey was distributed through institutional intermediaries and the number of eligible professionals who received the invitation was unknown, a response rate could not be calculated. Similarly, because hospital affiliation was intentionally not recorded to preserve anonymity, the number of participants recruited from individual hospitals could not be determined. A total of 8266 nurses and midwives completed the online questionnaire administered through Google Forms between 29 May and 8 July 2025.

### 2.4. Settings and Sample

The present study represents a secondary analysis of this national survey and was restricted to registered nurses working in AICUs of Romanian public hospitals (N = 1001). However, because hospital affiliation was intentionally not recorded in the questionnaire to preserve anonymity, hospital-level analyses were not possible. According to the inclusion criteria, participants had to be employed as AICU nurses in a Romanian public hospital and complete the online questionnaire. Nurses and midwives working in other healthcare settings or private institutions were not included in the present analysis. Eight participants had missing data for the Job Demands Index. Because the proportion of missing data was minimal (0.8% of the AICU sample), mediation analyses were conducted using complete-case analysis, resulting in an analytical sample of 993 participants.

### 2.5. Sample Size

The present analysis included all eligible AICU nurses identified in the nationwide survey. Because the study was based on an existing dataset, no a priori sample size calculation was performed. The final analytical sample comprised 993 participants.

### 2.6. Variables

#### 2.6.1. Sociodemographic Characteristics

The questionnaire collected information regarding participants’ gender, age, professional experience, marital status, number of children, educational level, and professional position.

#### 2.6.2. Job Demands

Job demands were operationalized as a cumulative exposure index summarizing six occupational demands frequently encountered in AICUs: night shifts, long work shifts, unscheduled call-ins to work, changes in work schedules, interactions with aggressive patients or family members, and negative emotional reactions to monitoring device alarms. The index was intended to quantify the overall burden of workplace demands rather than to represent a unidimensional latent construct. Consequently, the individual components were considered formative indicators contributing to an overall exposure score rather than interchangeable items of a psychometric scale. Each component was coded dichotomously, resulting in a cumulative score ranging from 0 to 6, with higher scores indicating greater exposure to workplace demands.

#### 2.6.3. Professional Fatigue

Professional fatigue was assessed using the following question: “To what extent do you feel tired after a working day?”. Responses were ranged on a five-point Likert scale from 1 (“not at all”) to 5 (“very high”).

#### 2.6.4. Depressive Symptoms

Depressive symptoms were assessed using the Romanian version of the 9-item PHQ-9 [15]. Each item is rated on a four-point Likert scale, producing a total score ranging from 0 to 27, with higher scores indicating greater symptom severity (0–4 = minimal or no depressive symptoms, 5–9 = mild, 10–14 = moderate, 15–19 = moderately severe, and 20–27 = severe) [16]. In the present sample, the Romanian version of the PHQ-9 demonstrated excellent internal consistency (Cronbach’s α = 0.925). Exploratory factor analysis using Principal Axis Factoring supported a unidimensional structure explaining 58.95% of the total variance. All PHQ-9 items were configured as mandatory fields in the online questionnaire. Consequently, no item-level missing data occurred for this instrument, and no imputation procedures were required.

#### 2.6.5. Turnover Intention

Turnover intention was assessed using the question: “Do you intend to leave your current workplace within the next 12 months?”, with a dichotomous response (Yes/No).

### 2.7. Data Analysis

The statistical IBM software SPSS 31.0 [17] and PROCESS macro, version 5.0 [18], were used for data analysis. The outcome was the turnover intention, the job demands were used as an independent variable, and professional fatigue and depressive symptoms were used as mediating variables. The categorical variables were described according to frequency and percentage and the continuous variables according to mean (m), standard deviation (SD), and the values of skewness and kurtosis, as indicators of a normal univariate distribution. Afterwards, the independent *t* test and chi-square test were used to compare the differences in levels of job demands, professional fatigue, and depressive symptoms between the respondents who had turnover intention and those who did not.

To test the indirect effect of job demands on the binary outcome, mediation analysis was performed using PROCESS macro (Model 4). In the primary model, job demands were specified as the independent variable (X), professional fatigue and depressive symptoms as parallel mediators (M1 and M2), and turnover intention as the dichotomous outcome variable (Y). A sensitivity analysis was subsequently conducted using the same mediation model after adjusting for demographic and professional covariates (age, gender, years of professional experience, education level, managerial position, and marital status). Because the dependent variable was binary, PROCESS estimated the final stage of the model using logistic regression, whereas all other paths were estimated using ordinary least squares regression.

The indirect effects were evaluated using percentile bootstrap confidence intervals based on 5000 resamples, which provided a robust estimate of mediation without assuming the normality of the indirect effect distribution. The mediation was considered statistically significant when the 95% bootstrap confidence interval did not include zero. The analysis produced estimates of the total effect, direct effect, and indirect effect, expressed in a log-odds metric, following an approach for models with dichotomous outcomes [19].

Prior to the analysis, the variables were examined for missing data and distributional properties; no violations requiring corrective transformation were identified. All the estimates were computed using two-tailed significance tests with α = 0.05.

The primary statistical analyses were specified *a priori* based on the study objectives, the proposed conceptual framework, and the predefined hypotheses regarding the direct and indirect associations between job demands, professional fatigue, depressive symptoms, and turnover intention. Additional sensitivity analyses adjusted for covariates were conducted during the peer-review process in response to reviewers’ comments to assess the robustness of the findings.

### 2.8. Ethical Considerations

The study consisted of an anonymous and voluntary survey, addressed exclusively to nurses and midwives in Romania, with the aim of assessing professional fatigue and working conditions, in order to substantiate professional development measures and improve the work environment.

The study did not involve patients, clinical interventions, diagnostic or therapeutic procedures, the collection of biological samples, access to medical records or the collection of identifiable medical data.

The responses were analyzed without the possibility of identifying the participants, exclusively for statistical purposes.

The online questionnaire began with an information page describing the study objectives, voluntary participation, confidentiality, and the intended use of the data. Participants indicated their informed consent by voluntarily completing and submitting the questionnaire.

Because the survey was fully anonymous and no personally identifiable information (e.g., names, contact details, or employing institutions) was collected, participants could not be identified or contacted on the basis of their responses, including responses to the PHQ-9 item assessing thoughts of self-harm or suicide. Consequently, no individual follow-up or referral procedures could be implemented.

## 3. Results

### 3.1. Participant Characteristics

The average age of the participating nurses was 44.32 years, with an average professional experience of 18.12 years. Most of the participants were female (886, i.e., 88.5%), with postgraduate education (707, i.e., 70.6%), and occupying executive positions (952, i.e., 95.1%). A significant proportion of the nurses were married (706, i.e., 70.6%) and had one or two children (766, i.e., 76.6%). Overall, the sample was composed predominantly of experienced female nurses employed in non-managerial positions, reflecting the demographic profile of the Romanian AICU nursing workforce. For the detailed demographic and professional characteristics of the participants, see Table 1 below.

### 3.2. Job Demands, Professional Fatigue, Depressive Symptoms, and Turnover Intention

The mean ± SD of the job demands was 3.03 ± 1.37, and the mean ± SD for depressive symptoms was 12.44 ± 7.00, indicating moderate levels for both variables. The mean job demands score indicates that participants were exposed, on average, to approximately half of the workplace demands included in the composite index. The mean PHQ-9 score corresponds to a moderate level of depressive symptoms. Regarding professional fatigue, nearly two-thirds of the participants (62.7%) reported high or very high levels of professional fatigue after a working day, whereas 15.4% indicated an intention to leave their current workplace within the following 12 months. (Table 2).

### 3.3. Associations Between Study Variables

Preliminary association analyses revealed positive, significant associations between turnover intention and professional fatigue (χ^2^ = 62.52, *p* ˂ 0.001). Nurses reporting turnover intention also reported considerably higher levels of professional fatigue. In particular, 37.7% of nurses with turnover intention reported very high professional fatigue compared with only 12.9% among those without turnover intention. Conversely, medium or lower levels of fatigue were substantially more frequent among nurses who did not intend to leave their workplace (Table 3).

Romanian AICU nurses reporting turnover intention had significantly higher mean job demands scores (3.60 vs. 2.93) and higher depressive symptoms scores (16.23 vs. 11.75) than those who did not report turnover intention. These data point out that both occupational demands and depressive symptoms are associated with nurses’ intention to leave (job demands: t = 5.64, *p* ˂ 0.001; depressive symptoms: t = 7.50, *p* ˂ 0.001). (Table 4).

Table 5 presents the comparisons of sociodemographic and professional characteristics according to turnover intention. No significant differences were observed with respect to age, professional experience, gender, marital status, or managerial position (all *p* > 0.05). In contrast, turnover intention was significantly more common among nurses with higher education than among those with secondary education (19.0% vs. 13.9%, χ^2^(1) = 4.01, *p* = 0.045).

### 3.4. Mediation Analysis

Mediation analysis revealed that job demands significantly predicted both mediators, with higher job demands associated with increased levels of professional fatigue (b = 0.215, *p* < 0.001) and depressive symptoms (b = 2.051, *p* < 0.001). The regression coefficients indicate that increases in job demands were associated with higher levels of both mediators, although the association was numerically stronger for depressive symptoms than for professional fatigue. However, job demands explained a modest proportion of the variance in both professional fatigue (12.9%) and depressive symptoms (16.1%), suggesting that additional organizational and individual factors contribute to these outcomes. (Table 6).

In the logistic regression model predicting turnover intention, both mediators were significant positive predictors. Specifically, each one-unit increase in professional fatigue was associated with a 37.8% increase in the odds of reporting turnover intention (OR = 1.378, 95% CI: 1.044–1.820, *p* = 0.024), while each one-unit increase in depressive symptoms increased the odds by 6.6% (OR = 1.066, 95% CI: 1.033–1.101, *p* < 0.001). Job demands also retained a significant direct effect on turnover intention, with each additional job demand increasing the odds of turnover intention by 20.7% (OR = 1.207, 95% CI: 1.046–1.392, *p* = 0.010), indicating partial mediation (Table 7).

Bootstrap analyses (5000 samples) confirmed that the total indirect effect was statistically significant (b = 0.200, LLCI = 0.127, ULCI = 0.287). Both specific indirect effects were also statistically significant: the indirect effect through professional fatigue was b = 0.069 (LLCI = 0.005, ULCI = 0.137), whereas the indirect effect through depressive symptoms was b = 0.131 (LLCI = 0.067, ULCI = 0.206) (Table 8). These findings indicate that both mediators contributed independently to the association between job demands and turnover intention.

#### Sensitivity Analysis

To assess the robustness of the findings, an additional mediation analysis was conducted adjusting for demographic and professional characteristics (age, gender, years of professional experience, marital status, managerial position, and education level). Job demands continued to show significant positive associations with both professional fatigue (b = 0.211, *p* < 0.001) and depressive symptoms (b = 2.062, *p* < 0.001). The inclusion of demographic and professional covariates resulted in only slight increases in the coefficients of determination (professional fatigue: R^2^ = 0.139 vs. 0.129; depressive symptoms: R^2^ = 0.170 vs. 0.161), indicating that the explanatory power of the models remained largely unchanged after adjustment (Table 9). The direct association between job demands and turnover intention also remained statistically significant (b = 0.181, *p* = 0.015) (Table 10). Furthermore, both indirect effects remained significant after adjustment for the covariates, supporting the robustness of the proposed mediation model (Table 11).

## 4. Discussion

A first notable finding of the study was the relatively high proportion of nurses reporting turnover intention (15.4%). In the context of the persistent workforce shortages affecting the Romanian healthcare system, this finding reinforces concerns regarding the sustainability of the nursing workforce. Similar concerns have been reported internationally, where turnover intention has consistently been identified as an early indicator of actual nurse turnover and organizational instability [4].

Although exploratory subgroup analyses indicated a higher prevalence of turnover intention among nurses with higher education, no significant differences were observed across the remaining sociodemographic characteristics. These findings suggest that occupational and psychological factors may play a more prominent role than most demographic characteristics in explaining turnover intention in this sample.

The study also showed that Romanian AICU nurses reported high levels of professional fatigue and moderate levels of depressive symptoms. These findings are consistent with previous evidence indicating that nurses working in intensive care settings experience considerable physical and psychological strain. An integrative review published in 2014, based on studies published between 2002 and 2013, concluded that fatigue is highly prevalent among nurses working in acute care environments [20]. Likewise, Huang et al. [21] reported that approximately one-quarter of ICU nurses experience depressive symptoms, while Çelik et al. [22] demonstrated significant associations between fatigue, anxiety, depression, and sleep quality among intensive care nurses. Taken together, these findings suggest that the demanding nature of critical care nursing places healthcare professionals at increased risk of psychological distress.

Beyond describing these outcomes, the present study provides evidence regarding the mechanisms linking occupational demands to turnover intention. Specifically, job demands remained a significant direct predictor of turnover intention, supporting the first hypothesis (H1). This finding is consistent with previous studies showing that demanding working conditions—including shift work [23], workplace violence [24], bullying [25] or workplace gaslighting [26] and alarm-related stress [27,28]—increase nurses’ intention to leave their jobs.

Importantly, the findings also supported the proposed mediating role of professional fatigue and depressive symptoms. Although the direct association between job demands and turnover intention remained significant, both professional fatigue and depressive symptoms significantly mediated the association between job demands and turnover intention. This indicates that demanding working conditions are connected with nurses’ intention to leave not only because of the objective characteristics of the work itself, but also because they gradually impair physical and emotional well-being. The sensitivity analysis showed that adjusting for demographic and professional characteristics resulted in only marginal increases in the explained variance of professional fatigue and depressive symptoms, suggesting that the associations with job demands were robust and largely independent of the covariates included in the models.

Nevertheless, the finding of partial rather than complete mediation deserves consideration. Although professional fatigue and depressive symptoms explained a substantial part of the association between job demands and turnover intention, as we underlined above, the persistence of a significant direct effect points to the additional mechanisms that are likely involved. Previous research [12,26] indicates that organizational factors such as job satisfaction, organizational commitment, perceived organizational support, leadership quality, and staffing adequacy, as well as individual factors including work engagement, resilience, coping strategies, and moral distress, may also contribute to nurses’ turnover intention. These factors were beyond the scope of the present study but represent important directions for future research aimed at developing more comprehensive explanatory models.

The observed mediation pathways are also plausible from a psychological perspective. High job demands may gradually deplete nurses’ physical and emotional resources, leading to persistent professional fatigue. As fatigue accumulates, nurses may perceive themselves as less able to cope with increasing work demands, resulting in reduced motivation, diminished work satisfaction, and a greater willingness to consider leaving their current workplace. Depressive symptoms may represent a further stage of this process, reflecting a more generalized deterioration in psychological well-being that extends beyond work-related exhaustion. In this context, turnover intention may be interpreted not only as a career decision but also as an adaptive response aimed at reducing prolonged occupational strain and protecting psychological health.

Taken together, these findings suggest that professional fatigue and depressive symptoms should not be viewed as isolated mechanisms but as complementary psychological responses to demanding working conditions [19,20,29]. Although both mediators reflect impaired psychological functioning, they capture different aspects of nurses’ responses to occupational stress. Professional fatigue primarily reflects work-related exhaustion that develops during or immediately after work shifts, whereas depressive symptoms represent a broader psychological condition that may extend beyond the workplace [19,20]. The finding that both variables independently mediated the association between job demands and turnover intention suggests that demanding working conditions may increase nurses’ intention to leave through multiple, interconnected psychological pathways rather than through a single mechanism [29,30].

Although our findings support the mediating roles of both professional fatigue and depressive symptoms, previous research suggests that these psychological mechanisms may not operate uniformly across different healthcare settings. A multicenter study conducted among Chinese nurses [29] found that emotional exhaustion mediated the association between depression and turnover intention, whereas depression did not significantly mediate the relationship between emotional exhaustion and turnover intention, highlighting the importance of the conceptual ordering of variables. Furthermore, longitudinal evidence indicates that depressive symptoms may predict turnover intention without necessarily leading to actual turnover, whereas fatigue appears to be associated with both turnover intention and subsequent job leaving [30]. These discrepancies indicate that the mediating roles of professional fatigue and depressive symptoms are likely influenced by contextual factors, study design, and the operationalization of psychological constructs, underscoring the need for further longitudinal research.

All these results thus extend previous research by simultaneously examining professional fatigue and depressive symptoms within a single mediation model in a large national sample of Romanian AICU nurses.

### 4.1. Research Implications

Future research should further investigate longitudinal models in order to determine the causal relationships and to examine additional mediators or possible moderators of this relationship, so as to contribute to a better understanding of the phenomenon. As other previous studies pointed out, there are important work-environment as well as individual aspects capable of mitigating the negative effects of job demands, such as work collaboration [31], job satisfaction [32,33], work engagement [34,35]. Other studies [36,37] have proved that in order to reconsider their turnover intentions, nurses highly valued a safe work environment, as well as positive emotions at work, such as pride in their jobs and a sense of fulfilment [38].

### 4.2. Practical Implications

From a practical standpoint, these results emphasized the need for Romanian public hospitals with AICUs which intend to reduce personnel turnover rates to address job demands by improving the work schedule and workplace safety, making sure to monitor the levels of fatigue of the personnel, as well as mental health indicators, such as depressive symptom presence. Efforts aiming to reduce fatigue, promote well-being, and provide psychological safety and support may help mitigate depressive symptoms as other studies have already demonstrated [39] and, consequently, lead to a lower turnover intention.

These findings also suggest that interventions aimed exclusively at reducing workload may be insufficient if the resulting psychological consequences are not simultaneously addressed. Preventing professional fatigue and identifying depressive symptoms at an early stage may represent complementary strategies for improving nurse retention in high-demand clinical settings.

### 4.3. Limitations

The current study, while providing insights into the turnover intention of Romanian nurses in the AICU, acknowledges several methodological and contextual limitations that should be mentioned.

The cross-sectional design precludes conclusions regarding temporal or causal relationships among the study variables. Although the mediation analysis identified statistically significant indirect associations that are consistent with the proposed theoretical framework, the temporal ordering of job demands, professional fatigue, depressive symptoms, and turnover intention cannot be established. Longitudinal studies are needed to confirm the directionality of these relationships.

The study was based on a convenience sample of nurses who voluntarily completed an online questionnaire. Because the survey invitation was disseminated through nursing directors of healthcare institutions, the exact number of eligible professionals who received the invitation was unknown and a response rate could not be calculated. Consequently, the representativeness of the sample cannot be formally established, and some degree of self-selection bias cannot be excluded.

The sample was exclusively drawn from AICUs in public hospitals, and, consequently, the findings might not apply to other types of units or to private institutions.

A further limitation concerns the measurement of professional fatigue and turnover intention using single-item indicators. Although this approach was considered appropriate for the descriptive objectives of the national survey and helped reduce respondent burden, particularly in an online questionnaire containing 82 items, single-item measures do not capture the multidimensional nature of these constructs and do not allow the assessment of internal consistency. Consequently, the observed associations should be interpreted as conservative estimates of the relationships among the study variables, as measurement error may have attenuated the magnitude of some effects. Future studies should consider using validated multi-item instruments to obtain a more comprehensive assessment of these variables.

The measurement of job demands reflects a pragmatic choice made for the purposes of the national survey. Instead of employing a validated psychometric instrument, we constructed a formative composite index that summarized exposure to several workplace demands considered relevant to the target population. As the index was designed to represent cumulative exposure rather than a latent psychological construct, formal psychometric validation was not undertaken. Consequently, the findings should be interpreted as reflecting the combined influence of the specific workplace demands included in the index. Future studies should examine whether similar results are obtained using validated multidimensional measures of job demands.

The data of the study were based on self-reports of nurses and there might be a social expectation bias because the respondents could be inclined to report lower levels of depressive symptoms or turnover intentions.

The mediating-effect analysis shows how job demands influence turnover intention due to professional fatigue and depressive symptoms, but might ignore certain potentially mediating variables, as already mentioned in Section 4.1.

In addition, because national demographic and professional data for Romanian AICU nurses are not publicly available, the representativeness of the study sample could not be formally assessed.

## 5. Conclusions

This study contributes to a better understanding of the mechanisms underlying turnover intention among AICU nurses by integrating organizational and psychological perspectives. The findings support the value of considering both workplace demands and nurses’ psychological well-being when examining workforce retention in highly demanding clinical settings. These insights provide a stronger basis for future research and for the development of evidence-informed strategies aimed at sustaining the nursing workforce.

## Figures and Tables

**Table 1:** Sociodemographic and professional characteristics of the study participants (N = 1001).

Characteristics	n (%)
Gender	
Female	886 (88.5)
Male	115 (11.5)
Education	
Highschool	10 (1.0)
Post-secondary education	707 (70.6)
Bachelor’s degree	222 (22.2)
Master’s degree	60 (6.0)
PhD	2 (0.2)
Marital status	
Married	706 (70.5)
Single	227 (22.7)
Living with a partner	68 (6.8)
Children at home	
0	196 (19.5)
1	393 (39.3)
2	373 (37.3)
3	37 (3.7)
4	2 (0.2)
National Teritory Unit	
North-East	75 (7.5)
South-East	34 (3.4)
South-Muntenia	103 (10.3)
South-West Oltenia	52 (5.2)
West	143 (14.3)
North-West	190 (19.0)
Center	58 (5.8)
Bucharest–Ilfov	346 (34.5)
Position	
Managerial	49 (4.9)
Non-managerial	952 (95.1)
Characteristics	**m (SD)**
Age (years)	44.35 (8.50)
Professional experience (years)	18.12 (10.06)

Note. Continuous variables are presented as mean (SD); categorical variables as n (%).

**Table 2:** Descriptive statistics for job demands, depressive symptoms, professional fatigue and turnover intention.

	m	SD	Skewness		Kurtosis	
			Statistic	SE	Statistic	SE
Job demands (N = 993)	3.03	1.37	0.21	0.08	−0.47	0.15
Depressive symptoms (N = 1001)	12.44	7.00	0.01	0.08	−0.89	0.15
	Not at all	Little	Medium	High	Very high	
Professional fatigue (%) (N = 1001)	0.5	5.6	31.2	46.1	16.6	
	No		Yes			
Turnover intention (%) (N = 1001)	84.6		15.4			

Note. m = mean; SD = standard deviation; SE = standard error.

**Table 3:** Association between professional fatigue and turnover intention among Romanian AICU nurses (N = 1001).

Turnover Intention	Professional Fatigue					χ^2^ (df)	*p*
	Not at All	Little	Medium	High	Very High		
No	0.4	5.8	33.9	47.0	12.9	62.52 (4)	˂0.001
Yes	0.6	4.5	16.2	40.9	37.7		

Note. Values are presented as percentages. χ^2^ = Pearson chi-square statistic; dfs = degrees of freedom.

**Table 4:** Differences in job demands and depressive symptoms according to turnover intention (N = 1001).

	m	SD	t	*p*
**Turnover intention**	**Job demands**			
Yes	3.60	1.36	5.64	˂0.001
No	2.93	1.35		
	**Depressive symptoms**			
Yes	16.23	7.22	7.50	˂0.001
No	11.75	6.75		

Note. m = mean; SD = standard deviation. Group differences were examined using the independent-samples *t* test.

**Table 5:** Differences in sociodemographic and professional characteristics according to turnover intention (N = 1001).

		**Turnover Intention**		**t**	***p***
		**Yes**	**No**		
Age, m (SD)		43.55 (8.22)	44.50 (9.05)	1.27	0.20
Professional experience, m (SD)		17.97 (8.55)	18.15 (10.24)	0.217	0.83
		**Turnover intention**		**χ^2^ (df)**	***p***
		**Yes**	**No**		
Gender (%)	Female	14.8	85.2	2.13 (1)	0.14
	Male	20.0	80.0		
Marital Status (%)	Married	15.2	84.8	0.78 (2)	0.67
	Single	15.0	85.0		
	Living with a partner	19.1	80.9		
Educational level (%)	Secondary education	13.9	86.1	4.01 (1)	0.04
	Higher education	19.0	81.0		
Managerial position (%)	Managerial	10.2	89.8	1.06 (1)	0.30
	Non-managerial	15.7	84.3		

Note. m = mean; SD = standard deviation; χ^2^ = Pearson chi-square statistic; dfs = degrees of freedom. Group differences for age and professional experience were examined using the independent-samples *t* test.

**Table 6:** Regression coefficients for the effects of job demands on professional fatigue and depressive symptoms. (N = 993).

Mediator	b	SE	t	*p*	LLCI	ULCI	R^2^
Professional fatigue	0.215	0.018	12.09	˂0.001	0.180	0.250	0.129
Depressive symptoms	2.051	0.149	13.78	˂0.001	1.759	2.343	0.161

Note. b = unstandardized regression coefficient; SE = standard error; LLCI = lower limit of the 95% confidence interval; ULCI = upper limit of the 95% confidence interval.

**Table 7:** Logistic regression model predicting turnover intention (N = 993).

Predictor	b (Log Odds)	OR	95% CI for OR	SE	Z	*p*
Job demands	0.188	1.207	1.046–1.392	0.073	2.58	0.010
Professional fatigue	0.321	1.378	1.044–1.820	0.142	2.26	0.024
Depressive symptoms	0.064	1.066	1.033–1.101	0.017	3.87	<0.001

Note. b = logistic regression coefficient (log odds); ORs = odds ratios; 95% CI for OR = 95% confidence interval for odds ratios; SE = standard error; Z = Wald statistic.

**Table 8:** Mediating effects of job demands on turnover intention through professional fatigue and depressive symptoms (N = 993).

Effect Types	Effect	Boot SE	Boot LLCI	Boot ULCI
Total indirect effect	0.200	0.041	0.127	0.287
X → M1 → Y	0.069	0.034	0.005	0.137
X → M2 → Y	0.131	0.036	0.067	0.206

Note. Job demands (X); professional fatigue (M1); depressive symptoms (M2); turnover intention (Y). Boot SE = bootstrap standard error; boot LLCI = lower limit of the 95% bootstrap confidence interval; boot ULCI = upper limit of the 95% bootstrap confidence interval.

**Table 9:** Regression coefficients for the effects of job demands on professional fatigue and depressive symptoms, after adjustment for demographic and professional characteristics (N = 993).

Mediator	b	SE	t	*p*	LLCI	ULCI	R^2^
Professional fatigue	0.211	0.046	11.77	˂0.001	0.176	0.246	0.139
Depressive symptoms	2.062	0.150	13.72	˂0.001	1.767	2.356	0.170

Note. b = unstandardized regression coefficient; SE = standard error; LLCI = lower limit of the 95% confidence interval; ULCI = upper limit of the 95% confidence interval.

**Table 10:** Logistic regression model predicting turnover intention, after adjustment for demographic and professional characteristics (N = 993).

Predictor	b (Log Odds)	OR	95% CI for OR	SE	Z	*p*
Job demands	0.181	1.198	1.037–1.385	0.074	2.45	0.015
Professional fatigue	0.343	1.409	1.059–1.873	0.145	2.36	0.019
Depressive symptoms	0.062	1.064	1.029–1.099	0.017	3.67	˂0.001

Note. b = logistic regression coefficient (log odds); ORs = odds ratios; 95% CI for OR = 95% confidence interval for odds ratios; SE = standard error; Z = Wald statistic.

**Table 11:** Mediating effects of job demands on turnover intention through professional fatigue and depressive symptoms, after adjustment for demographic and professional characteristics (N = 993).

Effect Types	Effect	Boot SE	Boot LLCI	Boot ULCI
Total indirect effect	0.200	0.042	0.129	0.289
X → M1 → Y	0.072	0.034	0.008	0.143
X → M2 → Y	0.127	0.036	0.062	0.062

Note. Job demands (X); professional fatigue (M1); depressive symptoms (M2); turnover intention (Y). Boot SE = bootstrap standard error; boot LLCI = lower limit of the 95% bootstrap confidence interval; boot ULCI = upper limit of the 95% bootstrap confidence interval.

## Data Availability

The dataset used and analysed in the current study is available from the corresponding author on reasonable request.

## Supplementary Materials

The following supporting information can be downloaded at: https://www.mdpi.com/article/10.3390/nursrep16080264/s1, Supplementary Material S1: Original Romanian Version of the Study Questionnaire [40,41,42,43,44,45,46,47].

## Public Involvement Statement

There was no public involvement in any aspect of this research.

## Guidelines and Standards Statement

This manuscript was developed following the APA 7th edition Journal Article Reporting Standards for Quantitative Research (JARS–Quant).

## Abbreviations

The following abbreviations are used in this manuscript:

AICU(s)	Anaesthesia and Intensive Care Unit(s)
AET	Affective Events Theory
(Boot) LLCIs	(Bootstrap) Lower Confidence Intervals
(Boot) SE	(Bootstrap) Standard Error
(Boot) ULCIs	(Bootstrap) Upper Confidence Intervals
CI	Confidence Interval
EUPSF	European Patient Safety Foundation
ICU	Intensive Care Unit
JD-R	Job Demands–Resources (Model)
ORs	Odds Ratios
SD	Standard Deviation
