# Supplementary material for: Too Demanding to Stay? Fatigue and Depressive Symptoms as Mediators of Turnover Intention in Romanian Anaesthesia and Intensive Care Nurses: A Cross-Sectional Study
Source: Nurs Rep. 2026 Jul 30;16(8):264. doi: 10.3390/nursrep16080264 (PMC13516649; doi:10.3390/nursrep16080264)
Supplement: Supplementary file 1 [file nursrep-16-00264-s001.zip › nursrep-4377097-Supplementary S1.pdf]

CHESTIONAR PENTRU EVALUAREA OBOSELII PROFESIONALE

LA ASISTENȚII MEDICALI ȘI MOAȘELE DIN ROMÂNIA

Section 1 of 10

CHESTIONAR PENTRU EVALUAREA OBOSELII PROFESIONALE A ASISTENȚILOR MEDICALI ȘI MOAȘELOR DIN ROMÂNIA

Stimate coleg / stimată colegă,

Vă invităm să participați la acest studiu de cercetare, care este derulat de către **Ordinul Asistenților Medicali Generaliști, Moașelor și Asistenților Medicali din România (OAMGMAMR),** în cadrul parteneriatului cu **European Patient Safety Foundation (EUPSF)** și a **campaniei naționale Fighting Fatigue Together (Luptăm împreună împotriva Oboselii).**

Această inițiativă a OAMGMAMR vizează **identificarea nevoilor și problemelor asistenților medicali și moașelor din țara noastră, având ca scop îmbunătățirea siguranței și calității vieții profesionale și personale** a acestora.

Participarea dvs. la acest studiu implică să răspundeți la un set de itemi referitori la statutul dvs. socio-demografic și profesional, precum și la un al doilea set de itemi care au ca obiectiv **evaluarea nivelului și impactului oboselii profesionale.**

Răspunsurile dvs. vor fi prelucrate statistic și vor fi utilizate **în scopul identificării factorilor care contribuie la oboseala profesională,** precum și **a impactului oboselii asupra sănătății fizice și mintale, a performanței și a satisfacției** profesioniștilor din domeniul sănătății.

Rezultatele obținute în urma prelucrării statistice a datelor obținute vor fi utilizate pentru elaborarea și publicarea de articole științifice, precum și pentru **dezvoltarea de politici și strategii destinate îmbunătățirii condițiilor de muncă din instituțiile medicale și creșterii siguranței pacienților și a profesioniștilor la locurile lor de muncă.**

Completarea acestui chestionar anonim durează aprox. 20 minute și este voluntară.

De asemenea, **completarea chestionarului reprezintă acordul dvs. pentru participarea la studiu, pentru prelucrarea statistică a răspunsurilor furnizate și pentru utilizarea datelor obținute** în scopurile menționate. **Confidențialitatea informațiilor personale va fi respectată** în acord cu legislația în vigoare și cu principiile deontologiei cercetării.

Pentru întrebări și informații suplimentare vă puteți adresa d-nei Doina Carmen Mazilu, Vice-Președinte OAMGMAMR: mazilu.carmen@oammrbuc.ro , secretariat@oamr.ro

Mențiune: Vă rugăm să completați acest chestionar doar dacă sunteți asistent medical generalist, moașă sau asistent medical de altă specialitate!

SECȚIUNEA I.

DATE SOCIO-DEMOGRAFICE ȘI PROFESIONALE

1. Profesia:

*Dacă aveți **altă specialitate decât cele enumerate**, vă rugăm specificați la opțiunea Alta.*

- Asistent medical generalist
- Asistent medical de radiologie
- Asistent medical de laborator
- Asistent medical de farmacie
- Asistent medical de igienă și sănătate publică
- Asistent medical de balneofizioterapie/balneofiziokinetoterapie și recuperare
- Asistent medical de nutriție și dietetică
- Asistent medical de stomatologie
- Asistent medical igienist de cabinet stomatologic
- Asistent medico-social
- Moașă
- Alta:..

2. Funcția:

- De execuție
- De conducere

3. Genul:

- Feminin
- Masculin

4. Vârsta: .... ani

5. Nivelul studiilor:

*Selectați **nivelul cel mai înalt** de studii.*

- Liceu Sanitar
- Școală Postliceală Sanitară
- Facultate de Asistență Medicală / Moașe
- Masterat
- Doctorat

6. Sunteți înscris(ă) în prezent într-un program de formare profesională de lungă durată (studii universitare de licență, masterat, doctorat, etc.)?

- Da
- Nu

7. Vechimea ca asistent medical / moașă: .... ani

8. Starea civilă:

- Căsătorit(ă)
- Necăsătorit(ă)
- Parteneriat civil

9. Numărul copiilor: ....

Introduceți 0, în cazul în care nu aveți copii.

10. Intervalul de vârstă al copilului/copiilor minori:

*Selectați **toate variantele de răspuns care se aplică.***

- 0-5 ani
- 6-12 ani
- 13-18 ani
- Nu se aplică

11. Persoane vârstnice / cu nevoi speciale aflate în îngrijire:

- Da
- Nu

12.a. LOCALITATEA în care se află instituția în care vă desfășurați activitatea profesională: ....

12.b. JUDEȚUL în care se află instituția în care vă desfășurați activitatea profesională: ....

În cazul în care localitatea este București, vă rugăm să menționați sectorul.

13. Tipul instituției medicale în care vă desfășurați activitatea profesională (locul de muncă principal):

Dacă lucrați într-un alt tip de secție/departament de spital sau într-un alt tip de instituție *decât cele menționate mai jos, vă rugăm* ***să specificați tipul secției/departamentului/instituției*** *la opțiunea Other.*

- **Spital - Secție medicală** (de exemplu: Medicină Internă, Reumatologie, Cardiologie, Pneumologie, Hematologie, Endocrinologie, Gastroenterologie, Boli metabolice și diabet, Dermatologie, Alergologie, Nefrologie, Urologie, Neurologie, Boli neurovasculare, Psihiatrie, Pediatrie, Neonatologie, Obstetrică-Ginecologie, Recuperare medicală, Oftalmologie, ORL, Boli infecțioase, Geriatrie, Oncologie medicală, etc.)

- **Spital - Secție chirurgicală** (de exemplu: Chirurgie Generală, Ortopedie, Traumatologie, Chirurgie toracică, Chirurgie cardiovasculară, Neurochirurgie, Chirurgie plastică, Chirurgie endocrină, Chirurgie urologică, Chirurgie oncologică, Chirurgie pediatrică, Chirurgie oftalmologică, Chirurgie în ORL, Chirurgie în obstetrică-ginecologie, Chirurgie în transplant etc.)

- **Spital - Secție ATI**

- **Spital - Bloc Operator**

- **Spital - UPU / CPU**

- **Spital - Ambulator/Spitalizare de zi**

- **Spital - Farmacie**

- **Spital - Laborator de analize medicale**

- **Spital - Radiologie și Imagistică medicală**

- **Spital - Alt tip de secție / departament** (de exemplu: Sterilizare, Unitate de Transfuzii, Explorări funcționale, Cardiologie intervențională, Endoscopie bronșică/digestivă, Serviciu/Compartiment specializat de prevenire a IAAM, etc.)
- **Alta** ....

14. Numărul de locuri de muncă pe care le aveți în prezent: ....

15. Locul de muncă principal (norma de bază) este:

- În sistemul public
- În sistemul privat

16. Salariul primit la locul de muncă unde aveți norma de bază este:

- Foarte bun
- Bun
- Insuficient

17. Numărul de ore lucrate pe săptămână (în medie):

- < 40 ore
- 40-50 ore
- 50-60 ore
- > 60 ore

18. Graficul dvs. de lucru cuprinde cel mai frecvent:

*Selectați **o singură variantă de răspuns** din cele enumerate.*

*Menționați **alte variante de ture de lucru** la opțiunea Other, dacă este cazul.*

- **Ture de 6 / 7 ore - ture de zi**
- Ture de 6 / 7 ore - ture de zi și ture în weekend și sărbători legale
- Ture de 6 / 7 ore - ture de zi, ture de noapte și ture în weekend și sărbători legale

- **Ture de 8 ore - ture de zi**
- Ture de 8 ore - ture de zi și ture în weekend și sărbători legale
- Ture de 8 ore - ture de zi, ture de noapte și ture în weekend și sărbători legale

- **Ture de 8 ore și ture de 12 ore - ture de zi, ture de noapte și ture în weekend și sărbători legale**

- **Ture de 12 ore - ture de zi, ture de noapte și ture în weekend și sărbători legale**

- **Alt tip de ture** ....

19. Ați lucrat în ture de noapte în decursul ultimei luni?

- Nu
- Da

20. Ați avut, în decursul ultimei luni, situații în care durata turei s-a prelungit?

- Nu
- Da

21. Ați avut, în decursul ultimei luni, situații în care ați fost chemat(ă) de acasă pentru o tură suplimentară?

- Nu
- Da
- **22. Ați avut, în decursul ultimei luni, situații în care turele dvs. au fost modificate?** Nu
- Da

23. Numărul mediu de pacienți îngrijiți în timpul unei ture:

- < 5
- 5-10
- 11-20
- 21-30
- > 30

24. Durata deplasării de acasă până la locul de muncă / de la locul de muncă până acasă:

- < 15 minute
- 15-30 minute
- 31-45 minute
- 46-60 minute
- 61-90 minute
- > 90 minute

25.a. În ultimele 2 săptămâni:

De câte ori ați avut liber un interval mai mic de 9 ore între 2 ture? ....

Introduceți 0, în cazul în care nu ați avut un interval mai mic de 9 ore între 2 ture în ultimele 2 săptămâni.

25.b. În ultimele 2 săptămâni:

De câte ori ați avut liber un interval de cel puțin 24 de ore între 2 ture? ....

Introduceți 0, în cazul în care nu ați avut un interval de cel puțin 24 ore între 2 ture în ultimele 2 săptămâni.

26. Ați fost în concediu medical în ultimele 12 luni?

- Nu
- Da
  Dacă da, vă rugăm să menționați cauza la opțiunea Altă situație
- Altă situație ....

27. Câte zile de concediu de odihnă v-ați luat de la începutul anului? ....

- 0
- 1 - 5 zile
- 6 - 10 zile
- 11 - 15 zile
- 16 - 20 zile
- >20 zile

28. Planificarea anuală a zilelor de concediu de odihnă este respectată:

- Da
- Nu

  Dacă nu, vă rugăm să menționați cauza la opțiunea Altă situație
  Altă situație ....

29. Aveți zile de concediu de odihnă neefectuate din anul anterior?

- Nu
- Da

  Dacă da, vă rugăm să menționați cauza la opțiunea Altă situație
- *Altă situație* ....

SECȚIUNEA II.

DATE PRIVIND ACTIVITATEA PROFESIONALĂ

30. Cât de mulțumit(ă) sunteți de activitatea dvs. profesională?

- Foarte mulțumit(ă)
- Mulțumit(ă)
- Nici mulțumit(ă), nici nemulțumit(ă)
- Nemulțumit(ă)
- Foarte nemulțumit(ă)

31. Munca pe care o depun are un scop important:

- Complet neadevărat
- Parțial neadevărat
- Neutru
- Parțial adevărat
- Complet adevărat

32. În ce măsură credeți că activitatea dvs. de la locul de muncă face posibil să existe un echilibru optim între viața profesională și viața personală?

- În foarte mare măsură
- În mare măsură
- Moderat
- În mică măsură
- În foarte mică măsură

33. Activitatea dvs. profesională presupune respectarea unor termene stricte?

- Tot timpul
- Aproape tot timpul
- De multe ori
- Câteodată
- Niciodată

34. Activitatea dvs. profesională presupune îngrijirea unor pacienți agresivi sau comunicarea cu rude agresive ale pacienților?

- Nu
- Da

35. Ați fost expus la vreun episod de intimidare/hărțuire/amenințare/violență în ultimele 12 luni?

- Da, zilnic
- Da, săptămânal
- Da, lunar
- Da, de câteva ori
- Nu

36. În ce măsură considerați că activitatea dvs. profesională afectează:

a. Viața de acasă (ex. treburi casnice, îngrijirea copiilor, etc.)

- Deloc
- Moderat
- Foarte mult

b. Relațiile interpersonale

- Deloc
- Moderat
- Foarte mult

c. Viața socială (ex. ieșiri în oraș, vizite la prieteni/rude, etc.)

- Deloc
- Moderat
- Foarte mult

d. Alte activități/angajamente

- Deloc
- Moderat
- Foarte mult

37. Intenționați să părăsiți profesia de asistent medical / moașă în următoarele 12 luni?

- Nu
- Da

SECȚIUNEA III.

DATE PRIVIND SUPORTUL ASIGURAT DE ANGAJATOR LA LOCUL DE MUNCĂ

38. În instituția în care lucrați, există un plan de acțiune destinat prevenirii stresului profesional?

- Da
- Nu
- Nu știu

39. Credeți că instituția în care lucrați acordă suficient sprijin personalului pentru a preveni oboseala profesională?

- Da
- Nu

40. Ați beneficiat vreodată de un program educațional sau alt tip de suport care a avut ca scop reducerea impactului stresului de la locul de muncă?

- Da
- Nu

41. În instituția în care lucrați, există o procedură pentru abordarea cazurilor de intimidare/ hărțuire/ amenințare/ violență la locul de muncă?

- Da
- Nu
- Nu știu

42. În instituția în care lucrați, au loc întâlniri periodice unde angajații își pot exprima opiniile și îngrijorările privind siguranța pacienților și a profesioniștilor?

- Da
- Nu
- Nu știu

43. În instituția în care lucrați, există o reprezentare sindicală?

- Da
- Nu
- Nu știu

SECȚIUNEA IV.

DATE PRIVIND FACILITĂȚILE DE ODIHNĂ / REPAUS LA LOCUL DE MUNCĂ

44. La locul dvs. de muncă, există un loc destinat odihnei / relaxării personalului?

- Da
- Nu
- Nu știu

45. La locul dvs. de muncă, aveți acces la locul de odihnă / relaxare pentru a vă odihni în timpul turei de lucru?

- Da
- Nu
- Nu se aplică (nu există un astfel de loc)

46. La locul dvs. de muncă, cât de des ați folosit locul de odihnă / relaxare pentru a „trage un pui de somn” (de aprox. 15-20 minute) în timpul turei de noapte, după ce sarcinile dvs. profesionale au fost preluate de către un coleg?

- În majoritatea turelor de noapte
- În aproximativ jumătate din turele de noapte
- În mai puțin de jumătate din turele de noapte
- Niciodată
- Nu se aplică (nu există un astfel de loc)

47. La locul dvs. de muncă, există o cameră în care vă puteți odihni / dormi după tura de noapte?

- Da

- Nu
- Nu știu

48. La locul dvs. de muncă, ați utilizat camera destinată odihnei / somnului după tura de noapte?

- Da
- Nu
- Nu se aplică (nu există o astfel de cameră)

49. În timpul turei de lucru, puteți decide când să luați o pauză?

- Întotdeauna
- De cele mai multe ori
- Câteodată
- Foarte rar
- Niciodată

50. La locul dvs. de muncă, cât de des reușiți să luați pauză în timpul turei?

- Foarte des
- De multe ori
- Câteodată
- Foarte rar
- Niciodată

51. La locul dvs. de muncă, există o reglementare privind respectarea pauzei de masă?

- Da
- Nu
- Nu știu

52. La locul dvs. de muncă, cât de des reușiți să luați pauza de masă în timpul turei?

- De fiecare dată
- De cele mai multe ori
- Câteodată
- Foarte rar
- Niciodată

53. Considerați că volumul de muncă este distribuit echitabil în echipa dvs. de lucru?

- Da
- Nu

54. Cât de des primiți ajutor și suport din partea colegilor dvs.?

- Întotdeauna
- De cele mai multe ori
- Câteodată
- Foarte rar
- Niciodată

55. Cât de des primiți ajutor și suport din partea superiorului dvs. direct?

- Întotdeauna
- De cele mai multe ori
- Câteodată
- Foarte rar
- Niciodată

SECȚIUNEA V.

DATE PRIVIND ODIHNA ȘI SOMNUL

56. În ultimele 2 săptămâni:

a. M-am trezit simțindu-mă odihnit(ă)

- Întotdeauna
- De cele mai multe ori
- Câteodată
- Foarte rar
- Niciodată

b. M-am simțit vesel(ă) și bine-dispus(ă)

- Întotdeauna
- De cele mai multe ori
- Câteodată
- Foarte rar
- Niciodată

c. M-am simțit calm(ă) și relaxat(ă)

- Întotdeauna
- De cele mai multe ori
- Câteodată
- Foarte rar
- Niciodată

d. M-am simțit activ(ă) și viguros(ă)

- Întotdeauna
- De cele mai multe ori
- Câteodată
- Foarte rar
- Niciodată

57. În cazul în care aveți mai multe ture de noapte consecutive, cât de bine reușiți să dormiți între două ture?

- Excelent
- Foarte bine
- Bine
- Nu prea bine
- Deloc bine

58. Atunci când sunteți liber(ă), cât de des reușiți să dormiți în intervalul orar 23-7?

- De fiecare dată
- De cele mai multe ori
- Câteodată
- Foarte rar
- Niciodată

59. Atunci când sunteți liber(ă), cât de des reușiți să dormiți suficient și să vă treziți simțindu-vă complet odihnit(ă)?

- De fiecare dată
- De cele mai multe ori
- Câteodată
- Foarte rar
- Niciodată

60. <u>În medie,</u> câte ore reușiți să dormiți într-o perioadă de 24 ore? ....

61. <u>În medie,</u> câte ore de somn reușiți să dormiți în fiecare noapte? ....

62. Simțiți că aveți suficient timp pentru odihnă și recreere între ture?

- Da
- Nu

63. Considerați că în prezent aveți o problemă cu somnul?

- Nu
- Da
-

64. A fost nevoie să luați unele medicamente (prescrise de medic sau suplimente nutritive) pentru a putea dormi?

- Nu
- Da
  Dacă da, vă rugăm să menționați la opțiunea Și anume denumirea acestor medicamente
- *Și anume*...................................

SECȚIUNEA VI.

DATE PRIVIND NIVELUL, EFECTELE ȘI IMPACTUL OBOSELII PROFESIONALE

Oboseala profesională reprezintă un fenomen care apare după o perioadă prelungită sau intensă de activitate fizică sau intelectuală, care are ca rezultat desfășurarea cu dificultate a activității profesionale și afectarea negativă a stării de bine și siguranței profesioniștilor din domeniul sănătății. Cel mai frecvent este cauzată de lipsa somnului odihnitor și este caracterizată de dorința fiziologică crescută de a adormi, somnolență, stare de vigilență redusă și afectarea performanței neuro-comportamentale. Este asociată cu apariția sindromului de burnout, cu un risc crescut de erori medicale și accidente, precum și cu influențarea negativă a vieții personale a profesioniștilor.

65. Pe o scară de la 1 la 10, care este nivelul oboselii profesionale pe care o resimțiți în prezent? ....

Unde 0 = fără oboseală, 1-3 = oboseală ușoară, 4-6 = oboseală moderată și 7-10 = oboseală severă

66. În ce măsură resimțiți oboseala după o zi de muncă?

- Deloc
- Puțin
- Mediu
- Destul de mult
- Foarte mult

67. Cât de frecvent ați ațipit sau ați adormit în următoarele situații:

a. Stând și citind

- Niciodată
- Foarte rar
- Uneori
- Destul de des
- Foarte des

b. Urmărind un program TV

- Niciodată
- Foarte rar
- Uneori
- Destul de des
- Foarte des

c. În timpul unei perioade de repaus (ex. după servirea mesei)

- Niciodată
- Foarte rar
- Uneori
- Destul de des
- Foarte des

d. Participând la o ședință

- Niciodată
- Foarte rar
- Uneori
- Destul de des
- Foarte des

e. În timpul unei conversații

- Niciodată
- Foarte rar
- Uneori
- Destul de des
- Foarte des

f. Ca pasager, în timp ce erați într-o mașină sau mijloc de transport în comun

- Niciodată
- Foarte rar
- Uneori
- Destul de des
- Foarte des

g. ***Ca șofer****, în timp ce mașina era oprită în trafic*

- Niciodată
- Foarte rar
- Uneori
- Destul de des
- Foarte des
- Nu se aplică

68. În ce măsură simțiți că oboseala afectează:

a. Calitatea activității dvs. profesionale?

- Deloc
- Moderat
- Foarte mult

b. Siguranța actului medical?

- Deloc
- Moderat
- Foarte mult

c. Relațiile cu colegii dvs.?

- Deloc
- Moderat
- Foarte mult

d. Comunicarea cu pacienții îngrijiți?

- Deloc
- Moderat
- Foarte mult

69. Ați făcut vreodată o greșeală la locul de muncă despre care credeți că a fost cauzată de oboseală?

- Nu
- Da
  Dacă da, vă rugăm să menționați la opțiunea Și anume ce tip de greșeală ....

70. În ultimele 2 săptămâni, cât de des au intervenit următoarele probleme?

a. Scăderea interesului de a realiza anumite activități

- Niciodată
- În câteva zile
- În mai mult de jumătate din timp
- Aproape tot timpul

b. Sentimente de tristețe, deprimare sau deznădejde

- Niciodată
- În câteva zile
- În mai mult de jumătate din timp
- Aproape tot timpul

c. Dificultăți legate de somn (adormire dificilă / insomnie / somnolență)

- Niciodată
- În câteva zile
- În mai mult de jumătate din timp
- Aproape tot timpul

d. Senzație de oboseală sau lipsă de energie

- Niciodată
- În câteva zile
- În mai mult de jumătate din timp
- Aproape tot timpul

e. Apetit redus sau alimentație excesivă

- Niciodată
- În câteva zile
- În mai mult de jumătate din timp
- Aproape tot timpul

f. Stare de neliniște, îngrijorare, nervozitate, iritabilitate, tensiune, anxietate sau agitație excesivă

- Niciodată
- În câteva zile
- În mai mult de jumătate din timp
- Aproape tot timpul

g. Dificultăți de concentrare / reamintire / memorie / gândire clară

- Niciodată
- În câteva zile
- În mai mult de jumătate din timp
- Aproape tot timpul

h. Sentimente negative

- Niciodată
- În câteva zile
- În mai mult de jumătate din timp
- Aproape tot timpul

i. Gânduri de suicid

- Niciodată
- În câteva zile
- În mai mult de jumătate din timp
- Aproape tot timpul

71. Ați apelat vreodată la consum excesiv de alcool pentru a reduce din impactul stresului?

- Nu
- Da

72. Ați fi reticent(ă) în a cere ajutor specializat (ex. consiliere psihologică) ca soluție de combatere a stresului?

- Nu
- Da

SECȚIUNEA VII.

DATE PRIVIND CAUZELE OBOSELII PROFESIONALE

73. Din punctul meu de vedere, **cauzele oboselii profesionale sunt reprezentate de**:

*Selectați **toate variantele de răspuns care se aplică**.*

*Menționați **alte cauze** la opțiunea Other, dacă este cazul.*

- Volumul mare de muncă
- Lipsa de personal
- Turele frecvente
- Timpul insuficient pentru odihnă
- Dificultatea de a lua pauze
- Responsabilitatea de a supraveghea colegi debutanți
- Sarcinile administrative excesive
- Condițiile de muncă inadecvate
- Atmosfera stresantă de la locul de muncă
- Presiunea din partea superiorilor
- Solicitările multiple simultane din partea medicilor
- Așteptările exagerate ale pacienților / familiilor
- Teama de malpraxis
- Altele: ....

74. Care credeți că este <u>principalul factor</u> care <u>contribuie în mod direct</u> la apariția oboselii profesionale? ....

Vă rugăm să menționați un singur factor!

75. Sunetele produse de alarmele aparatelor de monitorizare îmi provoacă o stare de stres suplimentar:

- Da
- Nu
- Nu se aplică

76. Efectele negative produse de alarmele aparatelor de monitorizare asupra mea includ:

Selectați ***toate variantele de răspuns care se aplică****.*

Menționați ***alte efecte*** *la opțiunea Altceva, dacă este cazul.*

- Stare de nervozitate crescută
- Reducerea nivelului de concentrare și de atenție
- Întreruperi în fluxul normal al activităților
- Disconfort
- Dureri de cap
- Tulburări de somn
- Scăderea performanței profesionale
- **Altceva** ....
- Nu mă deranjează alarmele
- Nu se aplică (nu sunt aparate de monitorizare în secție)

77. Acțiunile mele în legătură cu alarmele aparatelor de monitorizare includ:

Selectați ***toate variantele de răspuns care se aplică.***

Menționați ***alte acțiuni*** *la opțiunea Altceva, dacă este cazul.*

- Cresc limitele alarmelor la începutul turei
- Re-ajustez limitele alarmelor, atunci când acestea mă deranjează
- Ajustez volumul alarmelor
- În tura de noapte, anulez alarmele aparatelor
- Imediat ce aud o alarmă, verific starea pacientului
- Atunci când alarmele se aud în mod repetat, devin indiferent(ă) la acestea
- **Other** ....
- Nu se aplică (nu sunt aparate de monitorizare în secție)

SECȚIUNEA VIII.

DATE PRIVIND POSIBILELE SOLUȚII UTILE PENTRU REDUCEREA OBOSELII PROFESIONALE

78. Din punctul meu de vedere, măsurile care ar putea contribui la reducerea oboselii profesionale și la îmbunătățirea stării de bine a personalului sunt:

Selectați toate variantele de răspuns care se aplică.
*Menționați **alte măsuri** la opțiunea Other, dacă este cazul.*

- Reducerea numărului de ore lucrate
- Creșterea numărului de angajați
- Posibilitatea de a lua pauze mai lungi și mai frecvente
- Asigurarea suportului psihologic pentru personal
- Asigurarea accesului personalului la programe de gestionare a stresului
- Modernizarea echipamentelor și asigurarea adecvată a mentenanței tehnice
- Îmbunătățirea comunicării în echipa medicală și cu superiorii
- **Other** ....

79. Cât de des practicați activități fizice/sportive?

- Aproape zilnic
- De multe ori
- Uneori
- Foarte rar
- Niciodată

80. Cât de des practicați activități recreative?

- Aproape zilnic
- De multe ori
- Uneori
- Foarte rar
- Niciodată

81. Care este cea mai eficientă metodă pe care dvs. o utilizați pentru a combate oboseala profesională?

*Selectați **un singur răspuns** din cele menționate / enumerate mai jos!*

*Menționați la opțiunea Other **o altă metodă utilizată pentru a combate oboseala**, dacă aceasta nu este menționată în lista de mai jos.*

- Dorm/mă odihnesc
- Mă plimb
- Citesc
- Mă uit la filme
- Mă joc pe telefon
- Mă conectez la rețelele sociale
- Fac sport
- Beau apă
- Ascult muzică
- Consum cafea/energizante
- Mănânc ceva care îmi place
- Petrec timp cu familia
- Ies în oraș/socializez
- Mă relaxez
- Merg la un salon de înfrumusețare/masaj/etc.
- Plec în vacanță/concediu
- Practic hobby-uri (croșetat, grădinărit, etc.)
- Iau o pauză
- Învăț să spun nu
- **Other** ....

82. Dacă ați putea schimba un singur aspect pentru a vă îmbunătăți condițiile de muncă, care ar fi acesta?

*Selectați **un singur răspuns** din cele menționate / enumerate mai jos!*

*Menționați la opțiunea Other **un alt aspect pe care ați vrea să îl schimbați pentru a îmbunătăți condițiile de muncă**, dacă acesta nu este menționat în lista de mai jos.*

- Mai mult personal
- Comunicarea/colaborarea/relațiile de serviciu (cu șefii sau cu colegii)
- Împărțirea echitabilă a sarcinilor
- Planificarea/organizarea muncii
- Programul de muncă/reducerea orelor de muncă
- Ture de noapte mai puține
- Îmbunătățirea dotării cu aparatură/materiale
- Creșterea bugetului alocat instituției
- Conducerea/șefii
- Reducerea birocrației
- Reducerea numărului de pacienți

- Programe de suport/de consiliere/de reducere a stresului
- Evaluare psihologică
- Personal mai calificat/mai performant
- Pauze mai dese/respectarea orelor de pauză
- Reducerea stresului
- Reducerea nivelului de zgomot
- Să ies din sistem/să ies la pensie/reducerea vârstei de pensionare
- Alergatul prin spital
- Mărirea salariului
- Modificări legislative
- Sistemul de pază
- **Other** ....

Vă mulțumim pentru răspunsurile dvs.! Acestea sunt esențiale pentru crearea unui mediu de lucru mai sănătos, mai sigur și mai sustenabil!

Pentru a finaliza și trimite chestionarul, folosiți butonul "Submit" aflat în partea de jos a paginii!

REFERINȚE BIBLIOGRAFICE

- Batak T, Gvozdenović L, Bokan D, Bokan D. The impact of nurses' shift work on the fatigue level. South Eastern Europe Health Sci J 2013; 3(2): 120-7.
- Gander P, O'Keeffe K, Santos-Fernandez E, et al. Fatigue and nurses' work patterns: An online questionnaire survey. Int J Nurs Stud 2019; 98: 67-74.
- Gander P, O'Keeffe K, Santos-Fernandez E, et al. Work patterns and fatigue among DHB nurses. Executive Summary: 2016-2017 Nationwide Survey of DHB-Based Nurses Working in 6 Practice Areas. Massey University, University of New Zealand. Sleep/Wake Research Centre, February 2019. Disponibil la: https://www.safernursing24-7.co.nz/fileadmin/user_upload/downloads/Safer_Nursing_24-7_Survey_Report_Exec_Summary_FINAL_FOR_WEB_Feb_2019.pdf
- McClelland L, Holland J, Lomas JP, et al. A national survey of the effects of fatigue on trainees in anaesthesia in the UK. Anaesthesia 2017; 72(9): 1069-77.
- McClelland L, Plunkett E, McCrossan R, et al. A national survey of out-of-hours working and fatigue in consultants in anaesthesia and paediatric intensive care in the UK and Ireland. Anaesthesia 2019; 74(12): 1509-23.
- Podesta AM, Redfern N, Abramovich I, et al. Fatigue among anaesthesiologists in Europe: Findings from a joint EBA/NASC survey. Eur J Anaesthesiol 2024; 41(1): 24-33.
- Torabizadeh C, Yousefinya A, Zand F, et al. A nurses' alarm fatigue questionnaire: development and psychometric properties. J Clin Monit Comput 2017; 31: 1305-12.
- WHO/Europe Survey on the Mental Health and Well-Being of Health-Care Professionals (doctors and nurses) across the 27 European Union countries, Iceland and Norway. 2024. Disponibil la: https://www.who.int/europe/news-room/events/item/2024/10/22/default-calendar/who-europe-launches-first-of-its-kind-survey-on-mental-health-of-health-care-professionals
  https://www.eupsf.org/whomental-health-survey
  https://healthworkers-survey.ccomsuam.org/
- Wunderlich MM, Amende-Wolf S, Krampe H, et al. A brief questionnaire for measuring alarm fatigue in nurses and physicians in intensive care units. Sci Rep 2023; 13(1): 13860.

Dear colleague,
